# Flexible goal learning involves coordinated population activity in dCA1 and medial orbitofrontal cortex

**DOI:** 10.1371/journal.pbio.3003824

**Published:** 2026-05-29

**Authors:** Jiasong Li, Lingwei Tang, Xinhang Wei, Yumin Chen, Haibing Xu

**Affiliations:** Key Laboratory of Mental Health of the Ministry of Education, Guangdong‑Hong Kong‑Macao Greater Bay Area Center for Brain Science and Brain‑Inspired Intelligence, Department of Neurobiology, School of Basic Medical Sciences, Southern Medical University, Guangzhou, China; Center for Brain Research, Medical University of Vienna, AUSTRIA

## Abstract

Flexible goal‑directed navigation requires integrating changing goal information with a stable spatial map, yet how cortico-hippocampal circuits accomplish this remains unclear. We simultaneously recorded medial orbitofrontal cortex (mOFC) and dorsal CA1 (dCA1) while rats learned daily changing goal locations on a cheeseboard maze. Rats rapidly learned new goal locations and retained memory for them in the post‑probe session. Both regions contained goal‑related neuronal representations, but their profiles differed: dCA1 showed stronger spatial specificity, whereas mOFC showed more prominent learning‑related updating of goal‑related activity. Combining dCA1 and mOFC activity improved decoding of behavioral stage and learning block relative to either region alone, consistent with complementary contributions to ongoing behavior and learning state. Across learning, these population‑level differences were accompanied by stronger theta‑range synchronization and theta–gamma coupling during navigation than during goal periods. A recurrent network model with dynamic synaptic efficacy captured qualitative features of efficient acquisition and flexible goal updating, providing a candidate computational framework for how learning‑related temporal coordination could contribute to adaptive navigation.

## Abstract 2

How does the brain adapt when goals change? This study shows that coordinated neural rhythms between the medial orbitofrontal cortex and the hippocampus underlie flexible navigation, allowing animals to rapidly update their internal maps and switch between target locations.

## Introduction

The ability to navigate effectively toward a desired goal represents a fundamental cognitive function essential for survival. This process, known as goal‑directed navigation, is computationally demanding, requiring an organism to integrate multiple dynamic streams of information. These streams include its current spatial position [1,2], the value and location of its objective, and the sequence of actions needed to traverse the intervening space [3–5]. A hallmark of sophisticated navigation is flexibility, which is important for adapting to changing environments [6–8]. Unraveling the neural circuit mechanisms that support this flexibility remains a central challenge in systems neuroscience.

Decades of research have implicated a distributed network of brain regions in goal‑directed navigation, with the dorsal hippocampus and prefrontal cortex emerging as two particularly important components. The dorsal hippocampus, particularly its CA1 subfield (dCA1), is widely recognized for its central role in creating and maintaining a “cognitive map” of the environment [9–12]. This internal spatial representation is encoded by the activity of place cells that exhibit robust firing when an animal occupies a specific location—thereby providing an ongoing representation of the animal’s position [13–17]. In parallel, the medial orbitofrontal cortex (mOFC), a key subregion of the prefrontal cortex, has been strongly implicated in decision‑making and value‑based cognition [18–20]. The mOFC is thought to encode abstract task states, the expected outcomes of actions, and the subjective value of goals, thereby contributing to value‑guided choice behavior [21–24].

Indeed, studies have shown that OFC neurons can maintain goal‑related representations during navigation, potentially providing information about the animal’s intended destination [23,25]. While the canonical roles of dCA1 in spatial mapping (“where am I?”) and mOFC in goal representation (“where am I going?”) are well‑established, these prior findings make both regions strong candidates for supporting daily goal‑location learning. The precise nature of their interaction during flexible behavior remains poorly understood [26–28]. Functional interactions between hippocampal and orbitofrontal/prefrontal circuits are increasingly implicated in reward learning and memory‑guided behavior [29–31]. These observations, however, raise fundamental questions about the precise dynamics of their functional coordination. To address these questions, we performed large‑scale simultaneous in vivo recordings from dCA1 and mOFC while rats learned new reward locations each day in a cheeseboard maze task [32–34]. This design allowed us to examine how neural representations and inter‑regional coordination evolved from pre‑learning to post‑learning within the same daily session. We analyzed the data at multiple levels, including single‑neuron reorganization, mixed‑population decoding, oscillatory coordination, and short‑latency spike‑time coordination. Finally, we used a recurrent network model with dynamic synaptic efficacy to test whether an activity‑dependent interaction rule could, in principle, support efficient acquisition and flexible goal updating.

## Materials and methods

### Subjects

All procedures adhered to ethical guidelines and were approved by the Southern Medical University Experimental Animal Ethics Committee (L2019080). Five adult male Long‑Evans rats were implanted with 64 independently adjustable electrodes targeting the right dCA1 and mOFC. After a one‑week postoperative recovery, rats were maintained at 85% of their preoperative body weight with ad libitum access to water and a 12‑hour light/dark cycle. During the recovery period, electrodes were gradually advanced to the targeted regions. For the recording period, tetrode depths were fine‑adjusted in small increments between recording days to optimize unit isolation and maintain recording locations within the target regions.

### Surgical procedure

All animals were initially anesthetized in an acrylic chamber with 5% isoflurane at a flow rate of 1 L/min, and their heads were secured in a stereotaxic apparatus. During the surgery, 1%–3% isoflurane was delivered via a nose cone to maintain anesthesia, and body temperature was regulated with a heating pad. The target sites were measured and marked (dCA1: −3.6 mm AP, 2.0 mm ML; mOFC: 5.16 mm AP, 1.0 mm ML relative to bregma). The skull surface around the implantation sites was roughened to improve implant stability while avoiding interference with the goal areas. Vaseline was applied to seal around the craniotomy, and the entire implant was secured to the skull with dental acrylic. After surgery, the rats were returned to clean home cages with moist food pellets. For three days post‑surgery, meloxicam suspension (1 mg/kg, Boehringer Ingelheim) was added to the food to alleviate swelling‑related pain. Animals responded well to this treatment and resumed pre‑surgical activity levels within four days. No handling was performed during the first seven days post‑surgery.

### Behavioral tasks

Rats performed a daily goal‑location learning task on a circular cheeseboard maze, adapted from previous spatial memory paradigms [33,34]. The cheeseboard (150 cm in diameter, 1.5 cm in thickness) was made of plastic, painted white, and stood 55 cm above the floor in the recording room. The food wells were drilled into the surface of the maze in evenly spaced parallel rows and columns. A plastic‑made white start‑box (30 cm long, 20 cm wide, and 60 cm high) was placed along the edge of the board perpendicular to the rows of food wells. The top of the box was open to allow the tracking of the animal inside. Distal room cues were kept available throughout training and recording. Animals were maintained at approximately 85% of their free‑feeding body weight, and food reward consisted of pellets. Before formal training, rats were habituated to the cheeseboard for 30 min. They then underwent 3 d of visible‑reward training, during which food pellets were placed in the food wells together with nearby visible objects, allowing the animals to learn the basic task sequence of leaving the start box, locating and consuming two visually cued rewards, and returning to the start box. This was followed by hidden‑goal training without visible cues until the animals could reliably leave the start box, locate the two hidden reward sites, obtain the rewards, and return to the start box. Electrophysiological and behavioral recordings began only after animals had acquired this hidden‑goal version of the task.

On each recording day, animals were required to learn two rewarded goal locations. Reward locations varied across days to assess flexible updating of goal memory. Reward sites were selected according to the following constraints: the distance between the two rewarded locations on the same day was greater than 50 cm; the distance between each current‑day rewarded location and each rewarded location from the previous day was also greater than 50 cm; and all rewarded locations were positioned more than 20 cm away from the maze edge. Each daily session consisted of three consecutive sessions: pre‑probe, learning, and post‑probe. During the pre‑probe session (approximately 25 min), rats explored the maze without reward, allowing us to assess the relationship between neural activity and the previous day’s goal locations before new learning began. During the learning session, animals performed 40 trials to locate the two hidden rewarded sites and return to the start box. For analysis, the 40 trials were divided into four consecutive blocks of 10 trials each. During the post‑probe session (approximately 25 min), rats were again tested in the absence of reward to assess memory for the newly learned goal locations of that day. The order of these three sessions was identical across all recording days. Across the study, a total of 14 daily recording sessions were obtained from 5 rats (rat 1, n = 3; rat 2, n = 2; rat 3, n = 4; rat 4, n = 4; rat 5, n = 1). Unless otherwise noted, session‑level analyses in the manuscript refer to these daily recording sessions.

To minimize the influence of local olfactory cues, pellet dust was spread evenly across the cheeseboard surface throughout the task. In addition, after every 8 trials, the maze was wiped with 75% ethanol, briefly ventilated, and pellet dust was reapplied before the next set of trials began.

Animal position was monitored using an overhead video tracking system, and head coordinates (x,y) were extracted at 50 Hz using a DeepLabCut‑based tracking method. For each trial, trajectory distance was defined as the total path length from leaving the start box to returning to the start box, calculated from the tracked head positions. For probe‑session analyses, visits to reward locations were quantified using the same tracking coordinates. Circular regions of interest (ROIs; radius, 15 cm) were defined around each rewarded well. For reducing the influence of brief pass‑throughs and tracking noise, consecutive in‑ROI frames were merged into a single visit event, and two adjacent in‑ROI segments were further merged if the interval between them did not exceed 50 frames (approximately 1 s). The number of such merged ROI events within a probe session was defined as the number of goal visits. Visits to the previous day’s goal locations were quantified in the same way, except that the reward coordinates from the preceding recording session were used [33,34]. For within‑trial behavioral segmentation during the learning session, only two behavioral epochs were defined: Goal and Navigation. The Goal epoch was defined as the period during which the animal entered within 15 cm of a rewarded location and obtained the reward. The Navigation epoch was defined as the interval from leaving the start box to the first entry into the 15‑cm zone around a rewarded location, as well as the interval from leaving the first rewarded‑location area to entering the 15‑cm zone around the second rewarded location.

### Data acquisition and single‑unit recording

A 64‑channel headstage (RHD 64‑channel headstage) was used to continuously digitize electrophysiological data at a sampling frequency of 20 kHz. Raw data were processed using SpikeInterface [35] and Mountainsort4 [36], followed by manual sorting with phy software to isolate well‑separated units. Unit quality was evaluated by jointly considering spike waveform consistency, the spatial distribution of waveforms across channels, and cluster separation in feature space. We retained only units that showed stable waveform profiles, good cluster isolation, and clear refractory periods in the autocorrelogram and inter‑spike interval (ISI) histogram, with few or no spikes within the 0–1 or 0–2 ms range. For reproducibility and additional quality control, standardized unit‑quality metrics were further computed and recorded in CellExplorer (ProcessCellMetrics), including *refractoryPeriodViolation* (fraction of ISIs <2 ms), *isolationDistance*, and *lRatio*. Only units with a mean firing rate of at least 0.3 Hz were included in subsequent analyses.

Standardized unit features were then computed in CellExplorer, and putative cell types were assigned automatically based on spike waveform and autocorrelogram properties. Specifically, CellExplorer classifies putative cell types using the trough‑to‑peak latency of the spike waveform (*troughToPeak*) and the *acg_tau_rise* parameter obtained from triple‑exponential fitting of the autocorrelogram. Units with troughToPeak≤0.425 ms were classified as narrow‑waveform interneurons, whereas units with troughToPeak>0.425 ms and acg_tau_rise>6 ms were classified as wide‑waveform interneurons. All remaining units were classified as putative pyramidal cells (dCA1: 197 putative pyramidal cells and 75 putative interneurons; mOFC: 232 putative pyramidal cells and 53 putative interneurons).

### Histology and electrode location reconstruction

After the final recording session, rats were euthanized with an overdose of 5% isoflurane in an acrylic chamber, followed by cardiac perfusion with 0.9% saline (wt/vol), then with 4% formalin (wt/vol). Brains were extracted and stored in 4% formalin, then dehydrated in 5% sucrose solution. Once dehydrated, the brains were rapidly frozen and coronal sectioned (40 μm) using a cryostat, then stained with Cresyl Violet (Nissl stain). Sections containing electrode tracks were preserved, and final electrode positions were determined from digital images and records of daily electrode adjustments.

### Heatmap

For single‑neuron spatial analyses, the animal’s tracked head position was binned in two‑dimensional space to construct occupancy‑normalized firing‑rate maps. Position samples and spike times were synchronized and assigned to spatial bins of 5 × 5 cm. Occupancy maps were computed from the cumulative time spent in each bin, and spike maps were computed from the number of spikes emitted while the animal occupied each bin. Rate maps were obtained by dividing spike counts by occupancy on a bin‑by‑bin basis. Bins with occupancy below 200 ms were excluded, and the resulting maps were smoothed with a Gaussian kernel (kernel size = 5 bins, *σ* = 1.5 bins). Unless otherwise noted, only periods in which the animal was moving at >4 cm/s were included for spatial‑map analyses. In addition, only neurons whose spatial rate maps exhibited a peak firing rate >1 Hz were included in subsequent firing‑rate map analyses (dCA1: 191 putative pyramidal cells and 73 putative interneurons; mOFC: 229 putative pyramidal cells and 52 putative interneurons).

### Spatial information

Spatial information was calculated using the Skaggs information measure [37]:


$$\begin{array}{c} I=\sum_{i=\text{1}}^{N}p_{i}\frac{r_{i}}{\bar{r}}{\text{log}}_{\text{2}}\left(\frac{r_{i}}{\bar{r}}\right), \end{array}$$

where *p*_*i*_ is the occupancy probability of bin *i*, *r*_*i*_ is the firing rate in bin *i*, and $\bar{r}=\sum_{i=\text{1}}^{N}p_{i}r_{i}$ is the mean firing rate across all bins. Peak firing location was defined as the bin with the maximum firing rate in the smoothed rate map. The same procedure was used for both pre‑probe and post‑probe epochs.

### Goal‑related reorganization

To quantify goal‑related coding, reward locations from the previous day and the current day were aligned to the spatial firing‑rate map grid using the tracked behavioral coordinates. Because two reward locations were present on each day, overlap with previous‑day goals and current‑day goals was evaluated separately. For each neuron and each probe condition (pre‑probe or post‑probe), the high‑firing peak region was defined as all spatial bins in the smoothed rate map with firing rate greater than or equal to 80% of the peak firing rate of that map (FR≥0.8×max(FR)). For each reward location, a goal window was then constructed as a 3 × 3 square of spatial bins centered on the grid location of that reward site, corresponding to a 15‑cm neighborhood. The overlap score between the neuronal peak region and each goal window was defined as the number of bins shared by the two regions:


$$\begin{array}{c} \text{overlap}=|\text{peak bins}\cap \text{goal window}|. \end{array}$$

For each goal set (previous day or current day), overlap was computed separately for the two reward locations, and the larger of the two overlap values was taken as the overlap score for that goal set in that condition. A cell was considered to overlap with a given goal set when this overlap score was greater than zero.

Neurons were classified as goal‑related if they overlapped with the previous‑day goal set in the pre‑probe session or with the current‑day goal set in the post‑probe session. Among these goal‑related neurons, cells were classified as reorganization cells if they overlapped with the previous‑day goal set during the pre‑probe session and with the current‑day goal set during the post‑probe session. Those that did not show this pre‑to‑post updating pattern were classified as non‑reorganization cells.

To assess whether regional differences in the proportion of goal‑related neurons could be explained simply by differences in firing‑field size, we additionally quantified the size of the peak region for each neuron as the number of bins satisfying FR≥0.8×max(FR). As a continuous validation of the overlap‑based classification, we also quantified the distance between the firing‑field peak and the reward locations. Specifically, for each neuron and condition, the Euclidean distance from the peak bin to each of the two previous‑day goals and each of the two current‑day goals was calculated, and the minimum distance within each goal set was retained. These peak‑to‑goal distance measures were used to verify that cells classified as overlapping with a given goal set were located closer to that goal set in the corresponding probe condition. For region- and cell‑type‑level comparisons of goal‑related and reorganization‑defined cells, the fraction of classified cells was first computed within each recording session and then compared across sessions, in order to reduce confounding by variation in the total number of recorded units across sessions. Overall pooled proportions across all recorded units are shown in the corresponding pie charts as descriptive summaries only.

### Short‑latency spike–time interaction analysis

To quantify fine‑timescale spike–time coordination between simultaneously recorded neurons, we computed directed cross‑correlograms (CCGs) within each learning block. In these analyses, one neuron was treated as the reference neuron A, and the second neuron was treated as the target neuron *B*. Spike trains were converted to 1‑ms binary time series, in which each 1‑ms bin was assigned a value of 1 if it contained one or more spikes and 0 otherwise. For each ordered cell pair, CCGs were computed over a lag window of ±50 ms using 1‑ms bins. This analysis was interpreted as a measure of fine‑timescale functional coupling or excess coincidence, rather than direct evidence of monosynaptic connectivity.

For lag *τ*, the raw CCG counted the number of target‑neuron spikes occurring *τ* ms from each reference‑neuron spike. To reduce dependence on the total number of reference spikes, each CCG was normalized by the number of spikes emitted by the reference neuron:


$$\begin{array}{c} {\text{CCG}}_{\text{norm}}(\tau )=\frac{\text{CCG}(\tau )}{N_{A}}, \end{array}$$

where *N*_*A*_ is the total number of spikes emitted by neuron *A* within the analyzed block.

To reduce the contribution of slow co‑modulation and overall firing‑rate fluctuations, baseline coincidence was estimated as the mean normalized CCG across long‑lag bins at absolute lags of 20–50 ms and subtracted from the full CCG to obtain an excess‑coincidence function:


$$\begin{array}{c} {\text{CCG}}_{\text{excess}}(\tau )={\text{CCG}}_{\text{norm}}(\tau )-\text{baseline}. \end{array}$$

For each ordered cell pair, short‑latency coordination strength was quantified as the maximum excess coincidence within a predefined near‑zero‑lag window. The analysis window was set to 1–3 ms for within‑region pairs and 1–5 ms for cross‑region pairs.

To assess statistical significance, we generated an empirical null distribution using jittered surrogate spike trains. For each cell pair and each learning block, 1,000 surrogate datasets were generated by shifting every spike time of the target neuron B by an independently sampled integer offset drawn uniformly from [−25,25] ms, while approximately preserving coarse rate fluctuations. For each surrogate dataset, the same short‑latency coordination statistic was recomputed, yielding a null distribution against which the observed statistic was compared. The one‑sided empirical *p* value was calculated as


$$\begin{array}{c} p=\frac{\text{1}+\sum_{k=\text{1}}^{N_{\text{surr}}}\text{1}\left(T_{k}\geq T_{\text{obs}}\right)}{\text{1}+N_{\text{surr}}}, \end{array}$$

where *T*_obs_ is the observed short‑latency coordination statistic, *T*_*k*_ is the statistic from surrogate k, and $N_{\text{surr}}=\text{1},\text{0}\text{0}\text{0}$. Within each block, multiple pairwise tests associated with the same reference neuron were corrected using the Benjamini–Hochberg false discovery rate procedure (q<0.05). Cell pairs surviving this correction were classified as significant short‑latency interaction pairs.

To further characterize the overall direction of learning‑related changes in coordination strength for short‑latency interacting cell pairs, we performed a simple linear regression with block number as the predictor and coordination strength as the response variable, and used the resulting slope coefficient, β, as the trend metric: β>0 indicates an overall increasing trend in coordination strength, whereas β<0 indicates an overall decreasing trend. In parallel, we calculated the difference in coordination strength between Block 4 and Block 1, defined as Δ=B4−B1, to quantify the net end‑to‑start change. Cell pairs were classified only when the signs of β and Δ were consistent: pairs with β>0 and Δ>0 were defined as increased pairs, whereas pairs with β<0 and Δ<0 were defined as decreased pairs; all remaining pairs were left unclassified. This dual‑criterion scheme was used to require consistency between the overall across‑block trend and the net end‑to‑start change, thereby reducing misclassification arising from fluctuations in intermediate blocks.

For analyses relating pairwise temporal structure to reorganization status, each significant cell pair was assigned to the reorganization or non‑reorganization group according to the category of its first constituent neuron. The number of relevant cell pairs was first quantified within each session and then summarized at the session level for statistical comparisons, so as to reduce the risk of pseudo‑replication arising from treating individual cell pairs as independent samples.

### Assembly detection

Cell assemblies were detected to identify groups of neurons that repeatedly co‑fluctuated within short time windows during learning. In the present study, assembly detection was intended to capture population‑level co‑activation patterns rather than ordered spike sequences. Thus, the method identified sets of neurons whose binned activity rose and fell together within a fixed temporal window, and it was used to track the number, membership, and activation strength of these co‑active population patterns across learning.

To detect cell assembly patterns, we adopted an unsupervised statistical framework combining Principal Component Analysis (PCA) and Independent Component Analysis (ICA) [38,39]. Spike sequences were binned into 20 ms intervals to construct a spike‑correlation matrix for all neuron pairs. Assemblies were computed from principal components with eigenvalues exceeding the Marchenko–Pastur threshold. This method identifies fewer significant patterns than neurons, with each pattern explaining more variance than expected from independent neuron firing. Independent Component Analysis (FastICA) was then applied to calculate the weight vector for each assembly (component), i.e., the contribution of each neuron’s firing to the assembly. For assembly *i*, activation strength at time *t* was computed as


$$\begin{array}{c} S_{i}(t)=z(t{)}^{⊤}P_{i}z(t), \end{array}$$

where z(t) is the population activity vector at time t, and *P*_*i*_ is the projection matrix derived from the weight vector of assembly *i*. Discrete assembly activation events were defined as local peaks in $S_{i}(t)$ whose amplitude exceeded 5, following previous work [40]. The main diagonal was set to zero to ensure that isolated spikes of individual units did not contribute to *S*. Most detected assembly patterns consisted of a small number of neurons with high weights and a larger group of neurons with weights close to zero. To minimize contamination by ripple‑associated population bursts, time bins containing detected sharp‑wave ripple (SWR) events and the subsequent 100 ms period were excluded from the assembly analysis.

### Local field potential (LFP) analysis

Raw electrical signals were down‑sampled to 1,000 Hz. For each brain region, the LFP channel used for analysis was selected on the basis of histological verification confirming that the recording site was located within the target area (dCA1 or mOFC). Band‑pass filtering was performed using a fourth‑order Butterworth filter implemented with signal.butter and applied with zero‑phase forward‑backward filtering (signal.filtfilt) from the Python SciPy package. The frequency bands of interest were theta (4–12 Hz), low gamma (30–60 Hz), and high gamma (60–90 Hz). Power spectra and spectrograms were computed using the Welch method (scipy.signal.welch) with a 1‑s window and 90% overlap. For learning‑session analyses, power was first computed within each trial and then averaged across trials within each block (10 trials per block). Block‑level power values were then summarized at the session level for statistical comparison.

### Phase‑locking value (PLV)

Phase‑locking value (PLV) [41] is commonly used to measure the strength of rhythmic phase synchronization between brain regions. For each frequency band of interest, the LFP signals from dCA1 and mOFC were band‑pass filtered using a fourth‑order Butterworth filter (signal.butter and signal.filtfilt), and instantaneous phases $\phi_{\text{1}}(t)$ and $\phi_{\text{2}}(t)$ were extracted using the Hilbert transform (scipy.signal.hilbert). PLV was then computed as


$$\begin{array}{c} \text{PLV}=\left|\frac{\text{1}}{N}\sum_{t=\text{1}}^{N}e^{\;i(\phi_{\text{1}}(t)-\phi_{\text{2}}(t))}\right|, \end{array}$$

The value of *N* represents the length of the signal. The PLV value ranges from 0 to 1, where 1 indicates perfect synchronization and 0 indicates complete desynchronization.

### n:m phase synchronization

n:m phase synchronization [42] refers to cross‑frequency phase‑phase coupling, where the m ratio indicates the number of stable gamma oscillation cycles (*m*) corresponding to every *n* theta oscillation cycles. For a given pair of frequencies, the coupling strength was computed as


$$\begin{array}{c} r_{n:m}=\left|\frac{\text{1}}{N}\sum_{t=\text{1}}^{N}e^{\;i\left(n\phi_{\theta }(t)-m\phi_{\gamma }(t)\right)}\right|, \end{array}$$

where $\phi_{\theta }(t)$ and $\phi_{\gamma }(t)$ denote the instantaneous phases of dCA1 theta and mOFC gamma, respectively. For different integer ratios (e.g., 1:1, 1:2, …, 1:10), the distribution of $r_{n:m}$ values was calculated. Larger $r_{n:m}$ values indicate stronger cross‑frequency phase coupling. In the main analyses, n:m PLV was computed separately for dCA1 theta–mOFC low‑gamma and dCA1 theta–mOFC high‑gamma interactions during Goal and Navigation epochs.

### Theta phase locking of single units

Spike‑to‑theta phase locking was quantified using dCA1 theta as the reference rhythm, following previously established approaches [43]. For this analysis, LFPs were band‑pass filtered in the theta range (4–12 Hz). A tetrode located in the dCA1 was used as the reference theta phase, as theta phase has been reported to remain relatively constant above the CA1 cell layer [44]. Theta cycles were identified during periods when the rat’s speed exceeded 4 cm/s and theta power was greater than three times delta power. Each theta cycle was defined as the interval between 2 consecutive troughs of the theta wave. Theta periods were further restricted to segments in which no SWRs were detected on any CA1 tetrode using a 3 s.d. criterion. For each neuron, spikes were aligned to the reference theta phase to generate phase histograms spanning ${\text{0}}^{\circ }$ to ${\text{3}\text{6}\text{0}}^{\circ }$. Only neurons with at least 50 spikes in the theta‑aligned histogram were included. Deviations from a circular uniform distribution were tested using the Rayleigh test, and neurons with p<0.05 were classified as significantly phase‑locked. For significantly modulated neurons, the circular concentration parameter κ, which quantifies the strength of phase locking, was estimated by fitting the phase distribution with a von Mises distribution.

### Modulation index (MI)

To quantify phase–amplitude coupling between dCA1 theta and mOFC gamma, we computed the modulation index (MI) based on normalized entropy, following Tort and colleagues [45]. Let $x_{f_{p}}(t)$ denote the signal filtered in the phase‑frequency band and $x_{f_{a}}(t)$ denote the signal filtered in the amplitude‑frequency band. The instantaneous phase $\phi_{f_{p}}(t)$ was obtained from $x_{f_{p}}(t)$ using the Hilbert transform, and the amplitude envelope $A_{f_{a}}(t)$ was obtained from $x_{f_{a}}(t)$ using the Hilbert transform. The phase $\phi_{f_{p}}(t)$ was divided into 18 bins of ${\text{2}\text{0}}^{\circ }$ spanning ${\text{0}}^{\circ }$ to ${\text{3}\text{6}\text{0}}^{\circ }$, and the mean amplitude of $A_{f_{a}}(t)$ was computed within each phase bin. Let $P_{j}$ denote the normalized amplitude distribution across phase bins:


$$\begin{array}{c} P_{j}=\frac{\langle A_{f_{a}}\rangle_{\phi_{f_{p}}(j)}}{\sum_{j=\text{1}}^{N}\langle A_{f_{a}}\rangle_{\phi_{f_{p}}(j)}}, \end{array}$$

where N=18. Entropy was computed as


$$\begin{array}{c} H=-\sum_{j=\text{1}}^{N}P_{j}\text{log}P_{j}. \end{array}$$

The modulation index was then defined as


$$\begin{array}{c} \text{MI}=\frac{H_{\text{max}}-H}{H_{\text{max}}}, \end{array}$$

where $H_{\text{max}}=\text{log}(N)$ is the entropy of a uniform distribution. MI equals 0 when the amplitude distribution is uniform across phase bins, and larger MI values indicate stronger phase‑dependent modulation of amplitude.

### Population decoding

To quantify how neuronal population activity represented behavioral stage and learning progression, we used a multiclass decoding framework based on Gaussian naive Bayes (GaussianNB) implemented in scikit‑learn. Decoding analyses were performed separately for different neuronal populations, including all recorded neurons (ALL), dCA1‑only populations, mOFC‑only populations, and mixed populations generated by fixed-N replacement analyses. Before classifier training, all features were scaled using z‑score normalization.

For each learning trial, behavioral segmentation procedures were used to identify labeled task epochs (trace classes) and their temporal boundaries. For population decoding, each labeled behavioral epoch was segmented into consecutive non‑overlapping 400 ms windows, with each window treated as an individual sample. Within each sample, spikes from all quality‑controlled neurons in the decoding population were counted in 20 ms bins and converted into firing‑rate features. To ensure balanced training of the decoder, the number of samples was equalized across classes by randomly subsampling each class to match the smallest class. The concatenated firing‑rate values across neurons and bins were used as the feature vector for each sample. Decoding performance was evaluated using leave‑one‑trial‑out cross‑validation. In each iteration, one complete learning trial was held out as the test set, and all samples from the remaining trials were used for training. Thus, no time windows from the same trial were shared between the training and test sets, minimizing information leakage caused by temporal dependencies within individual trials. This procedure was repeated for all trials, and decoding performance was averaged across iterations to obtain the final decoding‑performance estimate for each analysis.

For behavioral‑stage decoding, only samples from the final learning block were used, and the classifier was trained to distinguish four classes: the two goal epochs and the two navigation epochs. For learning‑block decoding, neural activity samples from the Goal and Navigation epochs across all learning blocks were used separately, and the classifier was trained to distinguish the four learning‑block labels. Thus, both behavioral‑stage decoding and learning‑block decoding were implemented as four‑class classification problems.

In all decoding analyses, classifier performance was quantified using receiver operating characteristic (ROC) analysis of the class posterior probabilities generated by GaussianNB. True labels were first one‑hot encoded, and the predicted class‑probability matrix was obtained for the held‑out test set. To summarize multiclass decoding performance as a single quantity, we computed the micro‑average ROC curve by flattening the one‑hot label matrix and predicted‑probability matrix across classes into 1‑dimensional vectors and then calculating the ROC curve and its area under the curve (AUC). The resulting micro‑average ROC‑AUC was used as the primary decoding metric throughout the study. For the 4‑class decoding problems analyzed here, the chance level for ROC‑AUC was 0.5.

To test whether the decoding advantage of the combined population could be explained simply by increased neuron number, we performed fixed-N replacement analyses. In the primary analysis, the total number of neurons in the decoding population was held constant while a defined fraction of dCA1 neurons was randomly replaced with mOFC neurons. For each replacement fraction, mixed populations were generated by repeated random resampling, and the corresponding ROC‑AUC values were averaged across resamples. To test whether the effect depended on the direction of replacement, we also performed the reverse analysis, in which a fraction of mOFC neurons was replaced with dCA1 neurons under the same fixed-N procedure. For each replacement fraction, mixed populations were generated by repeated random resampling (n=200 resamples per fraction), which provided stable estimates of mean decoding performance for that replacement level.

To visualize the organization of population activity in a low‑dimensional state space, we additionally applied CEBRA to the same neural population datasets [46]. CEBRA embeddings were used only for visualization of behavioral‑stage and learning‑related population structure, whereas all quantitative comparisons reported in the main text were based on micro‑average ROC‑AUC from the GaussianNB decoder.

### Computational modeling architecture

To test whether a biologically plausible form of timing‑dependent synaptic efficacy could, in principle, support efficient goal learning and flexible updating, we constructed a recurrent neural network (RNN) model with two interacting subnetworks representing goal‑related and spatially structured components of the task. In this study, the model was used as a hypothesis‑generating consistency test rather than as proof of a specific physiological mechanism.

The model consisted of a reward subnetwork, corresponding functionally to mOFC‑like goal‑related signals, and a spatial subnetwork, corresponding functionally to dCA1‑like spatial signals. The reward subnetwork contained 4 reward‑input units projecting to 80 recurrent neurons (64 excitatory and 16 inhibitory). The spatial subnetwork contained 10 place‑input units projecting to 100 recurrent neurons (90 excitatory and 10 inhibitory). Excitatory neurons from both subnetworks projected to two readout units encoding movement velocity in the x- and y-directions. The agent therefore generated two‑dimensional movement as a function of the evolving network state.

To implement dynamic synaptic efficacy, we used a short‑term synaptic plasticity (STSP) rule based on the two‑variable model introduced by Mongillo and colleagues and used in related recurrent network studies [47]. For each presynaptic neuron j, synaptic efficacy was determined by the product of two variables: the fraction of available neurotransmitter $x_{j}(t)$ and the neurotransmitter utilization variable $u_{j}(t)$,


$$\begin{array}{c} S_{j}(t)=x_{j}(t)u_{j}(t). \end{array}$$

Let $r_{j}(t)$ denote the presynaptic activity of neuron j. The STSP variables evolved according to


$$\begin{array}{c} \frac{dx_{j}}{dt}=\frac{\text{1}-x_{j}(t)}{\tau_{x}}-u_{j}(t)x_{j}(t)r_{j}(t),\;\;\frac{du_{j}}{dt}=\frac{U-u_{j}(t)}{\tau_{u}}+U\left(\text{1}-u_{j}(t)\right)r_{j}(t), \end{array}$$

where $\tau_{x}$ is the recovery time constant of available neurotransmitter, $\tau_{u}$ is the time constant governing utilization, and U is the utilization increment parameter. Given the baseline synaptic weight $W_{j\to i}$, the instantaneous input from presynaptic neuron j to postsynaptic neuron i was


$$\begin{array}{c} I_{j\to i}(t)=W_{j\to i}\;x_{j}(t)\;u_{j}(t)\;r_{j}(t). \end{array}$$

Two classes of short‑term synaptic dynamics were implemented. Facilitating synapses were parameterized with $\tau_{x}=\text{2}\text{0}\text{0}$ ms, $\tau_{u}=\text{1}\text{5}\text{0}\text{0}$ ms, and U=0.15, whereas depressing synapses were parameterized with $\tau_{x}=\text{1}\text{5}\text{0}\text{0}$ ms, $\tau_{u}=\text{2}\text{0}\text{0}$ ms, and U=0.45. These parameter sets allowed synaptic efficacy to evolve dynamically as a function of recent presynaptic activity rather than remaining fixed over time.

The network‑controlled agent was embedded in a virtual navigation environment implemented using the RatInABox open‑source library [48]. The simulated task required the agent to navigate toward rewarded goal locations and was used to evaluate both initial acquisition and subsequent adaptation after a goal change. Three model variants were compared: (1) the full model with inter‑subnetwork connectivity and STSP, (2) a model with the same architecture but without plastic synaptic dynamics, and (3) a model without direct connections between the reward and spatial subnetworks. Parameters were updated with Adam optimizer. Gradients were masked according to the predefined connectivity structure and clipped by norm before each update.

Model performance was evaluated by quantifying trajectory efficiency and time required to reach the goal. To assess flexible updating, a model that had first been trained on one goal location was subsequently tested after the rewarded goal location was changed, and its adaptation was compared with that of a naive model without prior training. These analyses were used to determine whether dynamic synaptic efficacy within an interacting reward–spatial architecture could reproduce key qualitative features of efficient acquisition and flexible goal updating observed in the behavioral data.

### Speed controls

Because theta and gamma power can covary with locomotor behavior, we explicitly quantified the relationship between oscillatory power and instantaneous velocity. Velocity was derived from the tracked head position and aligned to the corresponding LFP samples. For the speed–power correlation analysis, power in the theta, low‑gamma, and high‑gamma bands was estimated in consecutive 200 ms windows, and each power estimate was paired with the mean locomotor velocity in the same time window within each session. To test whether learning‑related LFP changes could be explained by differences in locomotion across blocks, we focused on Navigation epochs and compared both mean navigation velocity and the distribution of navigation speeds between Block 1 and Block 4. For the distribution analysis, navigation velocities from 0 to 50 cm/s were binned in 2 cm/s intervals, and the z‑scored resulting binned distributions were then compared between Block 1 and Block 4.

### SWR detection and exclusion

SWR events were detected using custom Python code from ripple‑band filtered (150–250 Hz) LFP recorded on multiple dCA1 tetrodes, following previous approaches [49]. SWR events were identified when ripple‑band power exceeded 3 standard deviations above baseline. To avoid misclassifying high‑frequency fast‑gamma activity during locomotion as SWRs, detection was restricted to periods when running speed was <4 cm/s [50]. For analyses requiring exclusion of ripple‑related activity, periods containing detected SWRs were removed together with an additional 100 ms window following each detected event. In the present study, SWR‑related periods were excluded from the assembly analysis, the spike‑to‑theta phase‑locking analysis, PLV, PAC, and the cross‑correlogram‑based short‑latency coordination analysis.

### Breakpoint analysis

To quantify when behavioral and neural measures exhibited a transition during learning, we fit a continuous one‑breakpoint piecewise‑linear model to trial‑by‑trial data within each session. For each session, the variable of interest $y_{i}$ was modeled as a function of trial number *x*_*i*_ as


$$\begin{array}{c} y_{i}=\beta_{\text{0}}+\beta_{\text{1}}x_{i}+\beta_{\text{2}}(x_{i}-\tau {)}_{+}+\varepsilon_{i},\;\;(x_{i}-\tau {)}_{+}=\text{max}(\text{0},x_{i}-\tau ), \end{array}$$

where τ denotes the breakpoint trial, $\beta_{\text{1}}$ is the slope before the breakpoint, and $\beta_{\text{1}}+\beta_{\text{2}}$ is the slope after the breakpoint. Thus, $\beta_{\text{2}}$ represents the change in slope at the breakpoint.

The breakpoint τ was estimated by grid search across candidate trial values while excluding endpoint trials to ensure that observations were present on both sides of the breakpoint. For each candidate τ, model parameters $\left(\beta_{\text{0}},\beta_{\text{1}},\beta_{\text{2}}\right)$ were estimated by ordinary least squares, and the residual sum of squares (RSS) was computed. The candidate value that minimized the RSS was taken as the estimated breakpoint $\hat{\tau }$. From the fitted model, we extracted the breakpoint location, together with the pre‑breakpoint and post‑breakpoint slopes, for each session. Estimated breakpoint values and corresponding slopes were then summarized across sessions for statistical comparison.

### Statistical analyses

To reduce statistical non‑independence, continuous neural and behavioral metrics were computed within each recording session and then summarized at the session level for statistical comparison whenever possible.

For continuous variables, statistical comparisons were performed using nonparametric tests unless otherwise stated. Paired comparisons between two related conditions were assessed with the Wilcoxon signed‑rank test. Comparisons between two independent groups were assessed with the Mann–Whitney *U* test. Comparisons involving more than two independent groups were assessed with the Kruskal–Wallis test, whereas comparisons involving more than two related or repeated‑measures conditions were assessed with the Friedman test. When omnibus tests were followed by pairwise post hoc comparisons, Dunn’s multiple‑comparisons test was used as appropriate. For multiple comparisons, false discovery rate (FDR) was controlled using the Benjamini–Hochberg procedure, and adjusted *q* values are reported where indicated. For categorical outcomes, including comparisons of counts or proportions of classified cells, Fisher’s exact test was used.

For circular spike‑phase analyses, deviations from a uniform phase distribution were assessed using the Rayleigh test, as described above. For the short‑latency spike‑time interaction analysis, empirical *p* values were obtained from jittered surrogate distributions, and multiple testing across pairwise comparisons associated with the same reference neuron was controlled using the Benjamini–Hochberg false discovery rate procedure with q=0.05.

Unless otherwise noted, all statistical tests were two‑sided, and statistical results are reported with the exact test used, sample size (*n*), relevant test statistic, and exact *p* value or FDR‑adjusted *q* value where applicable. Classical statistical analyses and graph generation were performed in GraphPad Prism 9, whereas surrogate‑based analyses and custom computations were implemented in Python 3.8, as described above. Unless otherwise noted, all bar graph data are presented as mean ± SEM.

## Results

### Rats rapidly acquired new goal locations and exhibited goal‑related single‑neuron reorganization

**Fig 1:**
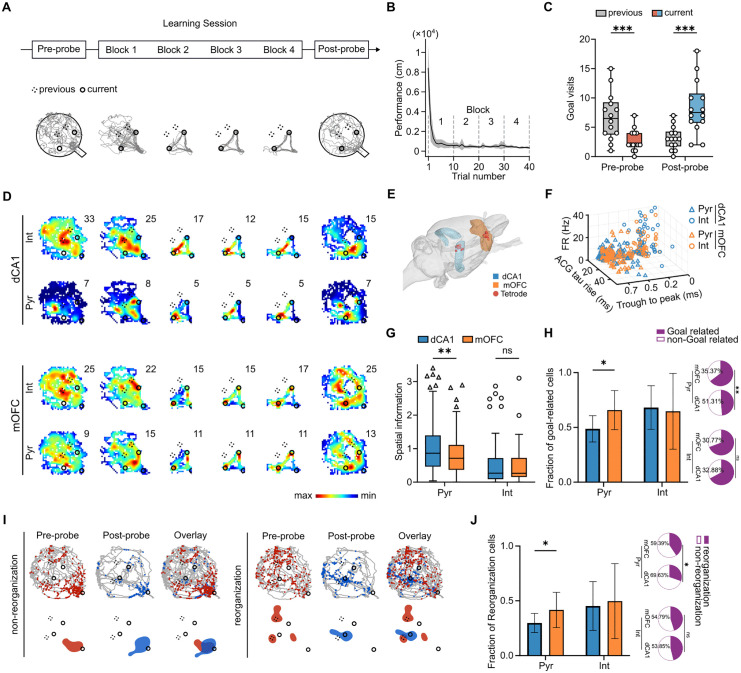
Behavioral performance and single‑neuron activity patterns during goal‑directed learning. **(A)** Experimental Paradigm. The top panel shows a schematic of the cheeseboard task, which consists of three sequential daily sessions: a pre‑probe session, a learning session, and a post‑probe session. The bottom panel shows a sample trajectory trace (gray line) and the two goal locations (black circles) for a single daily session. **(B)** The mean path length traveled by the rats across trials during the learning session. The black line represents the average path length over 14 sessions, with the gray shading indicating the standard error of the mean. The path length rapidly decreased, suggesting efficient learning of the new goal locations. **(C)** Comparison of the number of visited goal locations over the current and previous days during the pre- and post‑probe sessions. A significant increase in visits at the current day’s goal locations during the post‑probe session (Multiple Wilcoxon matched‑pairs signed‑rank tests with FDR correction, adjusted *q* values: pre‑probe, previous vs. current, *n* = 14 sessions, *W* = 91, *q* = 0.0002; post‑probe, previous vs. current, *n* = 14 sessions, *W* = −105, *q* = 0.0002), indicates that the rats formed a robust memory of the new goals. **(D)** Examples of single‑neuron firing rate heatmaps for different cell types in the medial orbitofrontal cortex (mOFC) and dorsal CA1 (dCA1). Heatmaps are shown for both pyramidal (Pyr) and interneurons (Int). The firing rate (FR) values are noted in the upper‑right corner of each heatmap. **(E)** The location of the electrode implantations in the brains of the rats. Red dots indicate the targeted recording sites in the right dCA1 and mOFC. **(F)** Visual examples of the distinct electrophysiological features used to classify neurons. Putative pyramidal neurons and interneurons in the mOFC and dCA1 are distinguished by their characteristic auto‑correlograms and spike waveforms. **(G)** Spatial information content of neurons in the mOFC (orange) and dCA1 (blue) (Mann–Whitney test, FDR‑corrected multiple comparisons, adjusted *q* values: Pyr, dCA1 vs. mOFC, *n*_dCA1_ = 197, *n*_mOFC_ = 232, *U* = 18,959, *q* = 0.0075; Int, dCA1 vs. mOFC, *n*_dCA1_ = 75, *n*_mOFC_ = 53, *U* = 1,923, *q* = 0.7577). **(H)** Session‑level fraction of goal‑related cells for putative pyramidal neurons (Pyr) and interneurons (Int) in dCA1 and mOFC (Multiple Wilcoxon matched‑pairs signed‑rank tests with FDR correction, adjusted *q* values: Pyr, dCA1 vs. mOFC, *n* = 14 sessions, *W* = −69, *q* = 0.0271; Int, dCA1 vs. mOFC, n = 14 sessions, *W* = 2, *q* = 0.9680). Pie charts at right show the corresponding overall fractions of goal‑related and non‑goal‑related cells across all recorded units and are included as descriptive summaries (Fisher’s exact test, Pyr, mOFC vs. dCA1, *n*_GoalmOFC_ = 148, *n*_non-mOFC_ = 81, *n*_GoaldCA1_ = 93, *n*_non-dCA1_ = 98, *p* = 0.0011; Int, mOFC vs. dCA1, *n*_GoalmOFC_ = 36, n_non-mOFC_ = 16, *n*_GoaldCA1_ = 49, *n*_non-dCA1_ = 24, *p* = 0.8477). **(I)** Schematic illustration of non‑reorganization and reorganization neurons. Top: animal path (gray), spikes locations (colored dots), and reward location (black circles) during task. Bottom: firing fields (colored region) of the same neurons. **(J)** Session‑level fraction of Reorganization cells for putative pyramidal neurons (Pyr) and interneurons (Int) in dCA1 and mOFC (Multiple Wilcoxon matched‑pairs signed‑rank tests with FDR correction, adjusted *q* values: Pyr, dCA1 vs. mOFC, *n* = 14 sessions, *W* = −77, *q* = 0.0271; Int, dCA1 vs. mOFC, *n* = 14 sessions, *W* = −12, *q* = 0.6273). Pie charts at right show the corresponding overall fractions of Reorganization and non‑Reorganization cells across all recorded units and are included as descriptive summaries (Fisher’s exact test, Pyr, mOFC vs. dCA1, *n*_ReorganizationmOFC_ = 93, *n*_non-ReorganizationmOFC_ = 136, *n*_ReorganizationdCA1_ = 58, *n*_non-ReorganizationdCA1_ = 133, *p* = 0.0322; Int, mOFC vs. dCA1, *n*_ReorganizationmOFC_ = 24, *n*_non-ReorganizationmOFC_ = 28, *n*_ReorganizationdCA1_ = 33, *n*_non-ReorganizationdCA1_ = 40, *p* > 0.9999). The underlying numerical data are provided in S1 Data.

To investigate the neural dynamics underlying flexible spatial learning, five male rats were trained on a goal‑directed navigation task within a 1.5‑m diameter cheeseboard maze (Fig 1A). Each daily session consisted of three distinct phases: a pre‑probe session, a learning session, and a post‑probe session [33,34]. The reward locations were changed daily to assess the flexibility of learning and memory. The learning session, which comprised 40 trials, required rats to navigate from a start box to locate two hidden reward locations and then return. Behavioral performance improved within the learning session, with trajectory length decreasing across trials (Fig 1B). To evaluate the time course of this improvement more explicitly, we performed trial‑resolved breakpoint analyses of trajectory length and found that the mean behavioral breakpoint occurred around trial 10, with a substantially steeper pre‑breakpoint slope than post‑breakpoint slope (S1H and S1I Fig), supporting the use of the first 10 trials as the early‑learning Block 1 while also indicating that the dominant reduction in slope occurred over the early part of the session [51–53]. Memory for the new goal locations was evident in the post‑probe session. Rats visited the current day’s reward locations more frequently than the previous day’s locations (Fig 1C), indicating memory for the newly learned goals.

We performed simultaneous in vivo multi‑channel electrophysiological recordings using a 64‑channel array of independently adjustable electrodes [32]. Thirty‑two electrodes targeted dCA1, and the remaining 32 targeted mOFC. Recording sites are shown in Fig 1E and were verified histologically (S7A Fig). Across 14 sessions, we isolated 272 neurons in dCA1, including 197 putative pyramidal neurons and 75 putative interneurons, and 285 neurons in mOFC, including 232 putative pyramidal neurons and 53 putative interneurons (Fig 1F). Consistent with the known functional specializations of these regions, putative pyramidal neurons in dCA1 exhibited significantly higher spatial information content and more spatially localized firing fields compared to those in mOFC (Fig 1G).

We next assessed how neural representations in these two areas adapted to the daily changes in goal locations by comparing the firing patterns between the pre‑probe and post‑probe sessions. Representative firing‑rate maps revealed goal‑related activity patterns in both regions (Fig 1D). We quantified this relationship by measuring the proportion of neurons whose firing fields overlapped with the reward locations. Cells classified as goal‑related exhibited overlap between their firing‑field peaks and goal locations, and this classification was further supported by a continuous peak‑to‑goal distance analysis rather than by an overlap measure alone (S4B Fig). Goal‑related neurons were identified in both dCA1 and mOFC, among both putative pyramidal neurons and interneurons (Fig 1H). To reduce confounding by differences in the total number of recorded units across sessions, we quantified the fraction of goal‑related cells within each session for each region and cell type. This session‑level analysis showed that the fraction of goal‑related cells was higher among putative pyramidal neurons in mOFC than in dCA1, whereas the fraction of goal‑related interneurons did not differ between the two regions (Fig 1H). This difference was not trivially explained by larger firing fields in mOFC, because the size of the 80% peak region did not differ between regions for putative pyramidal neurons (S4A Fig).

These analyses establish that daily learning was accompanied by goal‑related single‑neuron representations in both dCA1 and mOFC. On the basis of these probe‑dependent changes, neurons were subsequently classified into reorganization and non‑reorganization groups according to whether their goal‑related firing fields shifted between the pre‑probe and post‑probe sessions (Fig 1I). To account for variation in total unit yield across sessions, we quantified the fraction of reorganization cells within each session for each region and cell type. This session‑level analysis showed that the fraction of reorganization cells was higher among putative pyramidal neurons in mOFC than in dCA1, whereas the fraction of reorganization interneurons did not differ between the two regions (Fig 1J). Overall counts and proportions of goal‑related and reorganization‑defined cells are shown in the accompanying pie charts for descriptive reference. Thus, although both regions contained goal‑related neurons, learning‑related updating of goal‑referenced single‑neuron representations was more prominent among mOFC pyramidal cells. This observation is consistent with models proposing that prefrontal regions contribute goal‑related information relevant to route planning within a hippocampal spatial framework [23,54].

### Complementary roles of mOFC and dCA1 in spatial goal‑directed behavior

**Fig 2:**
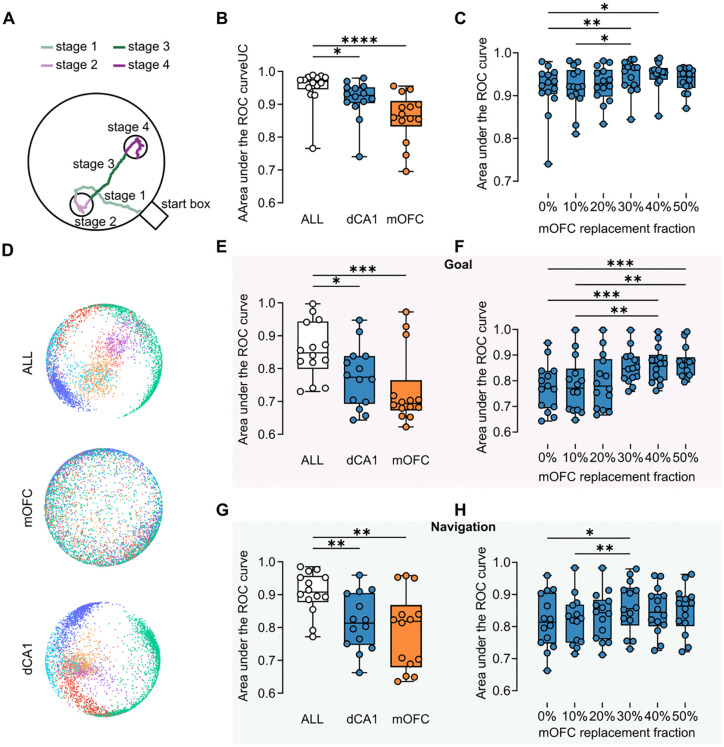
Complementary contributions of mOFC and dCA1 to spatial goal‑directed behavior. **(A)** Schematic Illustration of Behavioral Phases. This diagram divides a single trial into four distinct behavioral stages, each represented by a specific color: Stage 1 (light green), navigating from the start box to the first goal; Stage 2 (light purple), a stationary period at the first goal; Stage 3 (green), navigating from the first goal to the second; and Stage 4 (purple), a stationary period at the second goal. **(B)** Decoding of behavioral stages. The plots represent decoding performance quantified as micro‑average ROC‑AUC for three different neural populations: dCA1 alone (dCA1), mOFC alone (mOFC), and the combined dCA1–mOFC population (ALL) (Friedman test, Dunn’s multiple‑comparisons test, ALL vs. dCA1, *n* = 14 sessions, *Z* = 2.835, *p* = 0.0138; ALL vs. mOFC, *n* = 14 sessions, *Z* = 4.536, *p* < 0.0001; dCA1 vs. mOFC, *n* = 14 sessions, *Z* = 1.701, *p* = 0.2669). **(C)** ROC‑AUC comparisons across fixed-𝐍 mixing ratios in which 0%, 10%, 20%, 30%, 40%, or 50% of dCA1 neurons were replaced with mOFC neurons (Friedman test, Dunn’s multiple‑comparisons test, 0% vs. 30%, *n* = 14 sessions, *Z* = 3.347, *p* = 0.0082; 0% vs. 40%, *n* = 14 sessions, *Z* = 3.108, *p* = 0.0189; 10% vs. 30%, *n* = 14 sessions, *Z* = 2.869, *p* = 0.0412). **(D)** Representative examples of neural manifolds constructed from population activity. Different colors correspond to the behavioral phases defined in panel A. **(E, G)** ROC‑AUC for decoding learning block identity (Blocks 1–4) using neural populations during Goal epochs (E) and Navigation epochs (G), compared across ALL, dCA1, and mOFC populations (E: Friedman test, Dunn’s multiple‑comparisons test, ALL vs. dCA1, *n* = 14 sessions, *Z* = 2.835, *p* = 0.0138; ALL vs. mOFC, *n* = 14 sessions, *Z* = 3.969, *p* = 0.0002; dCA1 vs. mOFC, *n* = 14 sessions, *Z* = 1.134, *p* = 0.7705; G: Friedman test, Dunn’s multiple‑comparisons test, ALL vs. dCA1, *n* = 14 sessions, *Z* = 3.402, *p* = 0.0020; ALL vs. mOFC, *n* = 14 sessions, *Z* = 3.402, *p* = 0.0020; dCA1 vs. mOFC, *n* = 14 sessions, *Z* = 0.000, *p* > 0.9999). **(F, H)** ROC‑AUC for decoding learning block identity (Blocks 1–4) using neural populations during Goal epochs (F) and Navigation epochs (H), compared across fixed-𝐍 mixing ratios in which 0%, 10%, 20%, 30%, 40%, or 50% of dCA1 neurons were replaced with mOFC neurons (F: Friedman test, Dunn’s multiple‑comparisons test, 0% vs. 40%, *n* = 14 sessions, *Z* = 4.041, *p* = 0.0008; 0% vs. 50%, *n* = 14 sessions, *Z* = 4.142, *p* = 0.0005; 10% vs. 40%, *n* = 14 sessions, *Z* = 3.536, *p* = 0.0061; 10% vs. 50%, *n* = 14 sessions, *Z* = 3.637, *p* = 0.0041; H: Friedman test, Dunn’s multiple‑comparisons test, 0% vs. 30%, *n* = 14 sessions, *Z* = 3.030, *p* = 0.0366; 10% vs. 30%, *n* = 14 sessions, *Z* = 3.536, *p* = 0.0061). The underlying numerical data are provided in S2 Data.

Given the concurrent reorganization observed in both regions, we next asked whether combining mOFC and dCA1 population activity improved decoding of behavioral state. To test this, we used population firing data from mOFC alone, dCA1 alone, and the combined population during the consolidation phase (Block 4) to decode behavioral stage (Goal versus Navigation) (Fig 2A). The combined mOFC–dCA1 population yielded significantly higher decoding performance than either region alone (Fig 2B). To determine whether this combined‑population advantage could be explained simply by neuron number, we repeated the decoding analysis while holding the total population size constant and replacing a defined fraction of dCA1 neurons with mOFC neurons. Under this fixed-N framework, partial replacement improved decoding performance relative to the dCA1‑only baseline, with the largest improvement observed at intermediate replacement fractions (Fig 2C), indicating that the combined advantage cannot be explained by neuron count alone. This finding implies that a complete representation of the animal’s behavioral state is not fully contained within either region but emerges from their integrated activity. These results indicate that behaviorally relevant information was distributed across the two regions and was captured more effectively when their activity was analyzed jointly. Together with the fixed-N replacement analysis, this supports complementary rather than redundant contributions of mOFC and dCA1 to task‑state representation [55,56].

This combined‑population advantage was further illustrated using the unsupervised dimensionality reduction method CEBRA [46], which revealed that the neural manifold constructed from the joint population activity exhibited a clearer and more distinct separation of task stages compared to the manifolds from individual regions (Fig 2D). This visualization was consistent with the decoding analysis and suggested that combining activity from the two regions improved the separability of task‑related population states.

We next asked whether joint population activity also better captured learning‑related changes across the session. To do this, we separately analyzed neural activity during Goal and Navigation epochs and used it to decode learning block identity. In both behavioral contexts, decoding performance was higher for the combined dCA1–mOFC population than for either region alone (Fig 2E and 2G), indicating that learning‑related population changes were also more clearly represented when the two regions were considered together. We then repeated the block‑decoding analysis under the same fixed-N replacement framework. Replacing a fraction of dCA1 neurons with mOFC neurons improved block decoding during both Goal and Navigation epochs (Fig 2F and 2H), again indicating that mixed populations outperformed a single‑region population of matched size.

To test whether this result depended on the direction of replacement, we performed the reverse analysis by replacing mOFC neurons with dCA1 neurons. This manipulation likewise altered decoding performance and showed that the benefit of combining populations was not driven exclusively by one region (S2 Fig). Instead, the replacement analyses in both directions supported the view that dCA1 and mOFC make distinct but complementary contributions to the population representation of behavioral stage and learning progression. These results show that joint dCA1–mOFC activity provides a richer representation of both current behavioral state and within‑session learning stage than either region alone. Rather than indicating that one region dominates task coding, these findings support a distributed population code in which spatially informative dCA1 activity and goal‑related mOFC activity together improve decoding of task structure and learning state [55,56].

### State‑dependent oscillatory coordination between dCA1 and mOFC changed across learning

**Fig 3:**
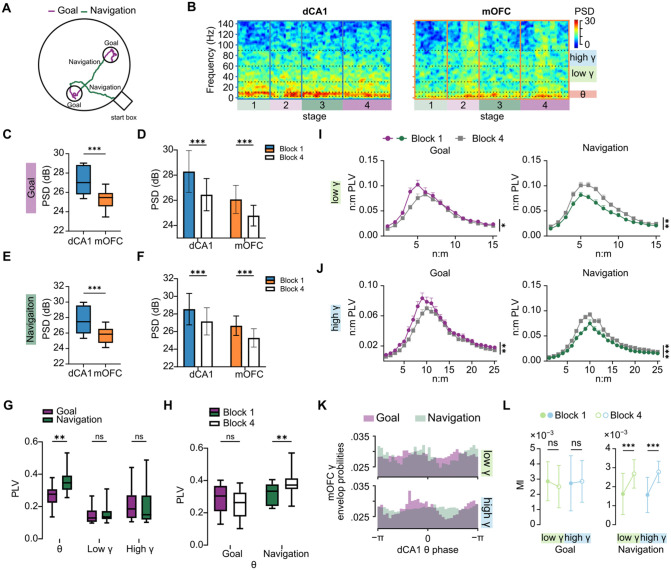
Dynamic neural oscillations accompany spatial goal‑directed learning. **(A)** Behavioral Classification. This schematic shows how the animal’s behavior was divided into two distinct phases for analysis: the Navigation phase (green), during which the animal traveled between goals, and the Goal phase (purple), defined as the period when the animal was within a 15 cm radius of a goal location. **(B)** Example power spectra from the mOFC and dCA1. The colored rectangles on the right y‑axis indicate the frequency bands of interest: theta (4–12 Hz), low gamma (30–60 Hz), and high gamma (60–90 Hz), which are analyzed in subsequent panels. **(C, E)** Theta power in Goal (C) and Navigation (E) phase in dCA1 and mOFC (C: Wilcoxon test, Goal, mOFC vs. dCA1, *n* = 14 sessions, *W* = −105, *p* = 0.0001; E: Wilcoxon test, Navigation, mOFC vs. dCA1, *n* = 14 sessions, *W* = −105, *p* = 0.0001). **(D, F)** Power changes during learning. These plots show the changes in theta power from Block 1 to Block 4 in the Goal epoch (D) and the Navigation epoch (F) for the mOFC and dCA1 (D: Multiple Wilcoxon matched‑pairs signed‑rank tests with FDR correction, adjusted *q* values: dCA1, Block 4 vs. Block 1, *n* = 14 sessions, *W* = 105, *q* = 0.0001; mOFC, Block 4 vs. Block 1, *n* = 14 sessions, *W* = 105, *q* = 0.0001; F: Multiple Wilcoxon matched‑pairs signed‑rank tests with FDR correction, adjusted *q* values: dCA1, Block 4 vs. Block 1, *n* = 14 sessions, *W* = 105, *q* = 0.0001; mOFC, Block 4 vs. Block 1, *n* = 14 sessions, *W* = 105, *q* = 0.0001). **(G)** Comparison of same‑frequency phase‑locking value (PLV) in the theta, low‑gamma, and high‑gamma bands between dCA1 and mOFC during Goal and Navigation epochs (Multiple Wilcoxon matched‑pairs signed‑rank tests with FDR correction, adjusted *q* values: θ, Goal vs. Navigation, *n* = 14 sessions, *W* = −97, *q* = 0.0017; Low *γ*, Goal vs. Navigation, *n* = 14 sessions, *W* = −51, *q* = 0.1200; High *γ*, Goal vs. Navigation, *n* = 14 sessions, *W* = 31, *q* = 0.2407). **(H)** Comparison of the PLV changes between early and late learning in Theta band of the dCA1 and mOFC (Multiple Wilcoxon matched‑pairs signed‑rank tests with FDR correction, adjusted *q* values: Navigation, Block 1 vs. Block 4, *n* = 14 sessions, *W* = −87, *q* = 0.0040; Goal, Block 1 vs. Block 4, *n* = 14 sessions, *W* = 49, *q* = 0.068). **(I)** Comparison of the PLV changes between early and late learning of the cross‑frequency n:m PLV between the dCA1 theta phase and the mOFC low‑gamma frequency, during Goal (left) and Navigation (right) (Wilcoxon test, Goal, Block 1 vs. Block 4, *n* = 14 sessions, *W* = −77, *p* = 0.0134; Navigation, Block 1 vs. Block 4, *n* = 14 sessions, *W* = 93, *p* = 0.0017). **(J)** Comparison of the PLV changes between early and late learning of the cross‑frequency n:m PLV between the dCA1 theta phase and the mOFC high‑gamma frequency, during Goal (left) and Navigation (right) (Wilcoxon test, Goal, Block 1 vs. Block 4, *n* = 14 sessions, *W* = −91, *p* = 0.0023; Navigation, Block 1 vs. Block 4, *n* = 14 sessions, *W* = 97, *p* = 0.0009). **(K)** Examples of the phase‑amplitude coupling (PAC) between the dCA1 theta rhythm and the mOFC gamma band. **(L)** Comparison of the modulation index during early and late learning for PAC of the mOFC low gamma‑dCA1 theta bands (left) and mOFC high gamma‑dCA1 theta bands (right) (Wilcoxon test, Goal Low γ, Block 4 vs. Block 1, *n* = 14 sessions, *W* = −17, *p* = 0.6257; Goal High *γ*, Block 4 vs. Block 1, *n* = 14 sessions, *W* = 5, *p* = 0.9032; Navigation Low *γ*, Block 4 vs. Block 1, *n* = 14 sessions, *W* = 101, *p* = 0.0004; Navigation High γ, Block 4 vs. Block 1, *n* = 14 sessions, *W* = 99, *p* = 0.0006). The underlying numerical data are provided in S3 Data.

Having found that joint mOFC–dCA1 activity improved decoding performance, we next examined whether LFP measures also revealed coordinated, state‑dependent dynamics between the two regions. We analyzed LFPs, which reflect the synchronized population‑level activity [57]. For these analyses, behavior was divided into Goal and Navigation epochs (Fig 3A). Spectral analysis revealed a clear dissociation in oscillatory profiles between the two regions (Fig 3B). Throughout the task, the dCA1 was dominated by low‑frequency activity, particularly in the theta band (Fig 3C and 3E), while the mOFC exhibited significantly stronger high‑frequency activity in the gamma band (S3A and S3B Fig). This pattern is consistent with the idea that dCA1 activity was more strongly dominated by theta‑range rhythms, whereas mOFC showed relatively stronger gamma‑range activity, in line with proposals that theta supports long‑range coordination and gamma reflects more local processing [58,59]. We then examined how these spectral features changed across learning. Trial‑resolved breakpoint analyses further showed that behavioral improvement and LFP‑related measures exhibited similar structured transition ranges across learning (S1F–S1I Fig), supporting the use of the first 10 trials as the early‑learning block. Theta power decreased from Block 1 to Block 4 in both dCA1 and mOFC during both Goal and Navigation epochs (Fig 3D and 3F). In addition, because theta and gamma power covary with behavioral state and locomotor variables [60–63], we explicitly tested whether the observed learning‑related LFP changes could be explained by trial‑to‑trial differences in movement. Navigation duration across learning and trial‑resolved locomotor profiles are shown in S1A and S1B Fig. Although power in several frequency bands was correlated with running speed, neither mean navigation velocity nor the distribution of navigation speeds differed between Block 1 and Block 4 (S1C–S1E Fig). Thus, the observed learning‑related spectral changes were not readily explained by differences in locomotor speed.

Because changes in power alone do not capture inter‑regional temporal coordination, we next examined oscillatory coupling between the two regions [64]. Phase–phase coupling analysis (PLV), which measures the consistency of timing between oscillations, revealed that theta‑band coherence between the mOFC and the dCA1 was significantly stronger during navigation compared to periods at the goal location (Fig 3G). Moreover, this navigational theta coherence increased significantly as learning progressed into the consolidation phase (Fig 3H), as well as in gamma band coherence (S3C Fig). These results indicate that inter‑regional theta coordination was stronger during navigation than during goal periods and became more pronounced later in learning. Together, they are consistent with a state‑dependent pattern of mOFC–dCA1 coordination that varies across task demands.

To understand how the rhythmic activity in the dCA1 might organize processing in the mOFC, we analyzed cross‑frequency coupling (CFC), such as Phase–amplitude coupling (PAC) and n:m PLV [65–67]. Epoch comparisons showed that n:m PLV between dCA1 theta and mOFC low- or high‑gamma activity did not differ significantly between Goal and Navigation epochs (S3E Fig). In contrast, PAC differed between Goal and Navigation for low‑gamma MI, whereas high‑gamma MI did not show a significant epoch difference (S3D Fig). However, learning‑related changes emerged across blocks, with both low‑gamma and high‑gamma coupling increasing from Block 1 to Block 4. By contrast, coupling during Goal epochs showed decrease across learning (Fig 3I and 3J). Notably, learning‑related enhancement of PAC was evident only during Navigation, with no changes during Goal epochs (Fig 3K and 3L). One possible interpretation is that, during navigation, dCA1 theta helps structure the timing of mOFC activity. This view is consistent with the stronger theta–gamma coupling observed during navigation and with prior work linking hippocampal–prefrontal theta coordination to active task performance [68,69].

To assess theta‑related spike organization at the single‑unit level, we quantified spike‑to‑theta phase locking in both regions. Theta phase‑coupled neurons were observed in both dCA1 and mOFC (S6A Fig). During Goal epochs, neither the proportion of phase‑coupled neurons nor phase concentration differed between Block 1 and Block 4 (S6B and S6C Fig). By contrast, during Navigation, both regions showed a higher proportion of theta phase‑coupled neurons in Block 1 than in Block 4, whereas phase concentration remained largely unchanged (S6D and S6E Fig). These results indicate that theta‑structured spiking was present in both regions, while also suggesting that learning‑related changes in population‑level inter‑regional coordination cannot be explained solely by an increased proportion of phase‑coupled neurons or by stronger single‑unit phase locking.

Because SWR events are enriched during goal‑related periods and could influence state‑dependent LFP measurements, we also quantified their occurrence across the task. SWRs were more frequent during Goal than during Navigation epochs, particularly in later blocks (S6F and S6G Fig). Together with the explicit treatment of SWR‑containing periods in the analysis pipeline, this control supports the interpretation that the main oscillatory findings reflect a broader state‑dependent reorganization of dCA1–mOFC coordination, rather than an artifact arising from ripple‑enriched goal periods.

### Learning‑related changes in short‑latency spike‑time coordination were linked to region‑specific neuronal reorganization

**Fig 4:**
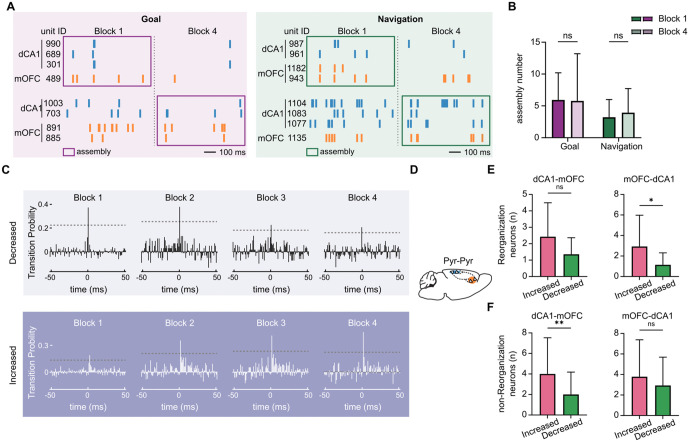
Learning‑related changes in short‑latency spike‑time coordination were linked to region‑specific neuronal reorganization. **(A)** Representative examples of neuronal assemblies detected in dCA1 and mOFC. Assemblies from both the Navigation and Goal phases are shown for Block 1 (early learning) and Block 4 (late learning). **(B)** Comparison of the number of mOFC–dCA1 assemblies identified during the Navigation and Goal phases in Block 1 and Block 4 (multiple Wilcoxon matched‑pairs signed‑rank tests with FDR correction, adjusted *q* values: Goal, Block 1 vs. Block 4, *n* = 14 sessions, *W* = 17, *q* = 0.6904; Navigation, Block 1 vs. Block 4, *n* = 14 sessions, *W* = −9, *q* = 0.6904). **(C)** Representative examples of cross‑regional short‑latency spike‑time coordination patterns that decreased or increased across learning. The top plot shows a pair with reduced excess coincidence from Block 1 to Block 4, whereas the bottom plot shows a pair with increased excess coincidence over the same interval. **(D)** Schematic illustration of the ordered cross‑regional pyramidal–pyramidal (Pyr–Pyr) cell‑pair analysis between dCA1 and mOFC. **(E)** Comparison of the number of reorganization neurons included in cross‑regional Pyr–Pyr pairs with decreased or increased short‑latency coordination for dCA1–mOFC (left) and mOFC–dCA1 (right) (Wilcoxon test, mOFC–dCA1, Decreased vs. Increased, *n* = 14 sessions, *W* = −46, *p* = 0.0439; dCA1–mOFC, Decreased vs. Increased, *n* = 14 sessions, *W* = −44, *p* = 0.0830). **(F)** Comparison of the number of non‑reorganization neurons included in cross‑regional Pyr–Pyr pairs with decreased or increased short‑latency coordination for dCA1–mOFC (left) and mOFC–dCA1 (right) (Wilcoxon test, mOFC–dCA1, Decreased vs. Increased, *n* = 14 sessions, *W* = −23, *p* = 0.3833; dCA1–mOFC, Decreased vs. Increased, *n* = 14 sessions, *W* = −66, *p* = 0.0078). The underlying numerical data are provided in S4 Data.

After observing dynamic interactions at the LFP level, we next investigated whether similar coordination changes occur at the cellular level. We first identified neuronal assemblies during Goal and Navigation epochs based on correlated patterns of ensemble activity [39]. This analysis was intended to assess whether learning‑related coordination changes were accompanied by broad reorganization of co‑active cross‑regional neuronal groups. Representative assemblies were observed in both regions during Block 1 and Block 4 (Fig 4A), and the number of assemblies did not significantly change from Block 1 to Block 4 (Fig 4B). We therefore hypothesized that learning‑related changes in this circuit may reflect reweighting of coordination within existing neuronal assemblies rather than recruitment of additional neurons.

To further assess these learning‑related timing changes, we used a cross‑correlation–based approach to quantify functional spike‑timing covariation between putative neuron pairs [70]. This metric reflects the probability that spikes in one unit tend to precede spikes in another within a short temporal window, without implying direct synaptic connectivity. An increase in spike‑timing covariation from Block 1 to Block 4 was interpreted as stronger short‑latency temporal coordination (Increased), whereas a decrease was interpreted as weaker coordination (Decreased) (Fig 4C). We focused first on cross‑regional pyramidal–pyramidal pairs, because these pairs showed the clearest learning‑related changes in short‑latency coordination. When we compared the number of reorganization neurons participating in pairs with increased versus decreased coordination, mOFC‑to‑dCA1 pyramidal pairs with increased coordination included a greater number of reorganization neurons than pairs with decreased coordination. But, the corresponding dCA1‑to‑mOFC comparison was not significant (Fig 4D and 4E). By contrast, for non‑reorganization neurons the opposite tendency was observed: dCA1‑to‑mOFC pyramidal pairs with increased coordination contained more non‑reorganization neurons than pairs with decreased coordination, whereas the mOFC‑to‑dCA1 comparison was not significant (Fig 4F). Detailed session‑wise counts of significant and total candidate cross‑regional pyramidal–pyramidal pairs underlying these comparisons are provided in S1 and S2 Tables.

This asymmetry suggested that learning‑related changes in cross‑regional short‑latency coordination were linked to different neuronal subpopulations in the two regions. In mOFC, increased coordination preferentially involved neurons whose firing fields had reorganized with the newly learned goal structure. In dCA1, by contrast, increased cross‑regional coordination more often involved neurons that retained non‑reorganization profiles. Within‑region analyses further refined this dissociation: in mOFC pyramidal–pyramidal pairs, increased coordination was enriched for reorganization neurons, whereas in dCA1 pyramidal–pyramidal pairs, decreased coordination was associated more strongly with non‑reorganization neurons (S5 Fig). Detailed session‑wise counts for the within‑region pyramidal–pyramidal pair analyses are provided in S3 and S4 Tables. Together, these results link learning‑related changes in short‑latency spike‑time coordination to probe‑defined reorganization of single‑neuron representations. Rather than reflecting diffuse changes across all neurons, the pairwise timing analysis suggests a region‑specific redistribution of short‑latency coordination: mOFC pyramidal neurons more often participated in reorganization‑linked timing changes, whereas dCA1 non‑reorganization neurons were associated with a different pattern across learning, including increased cross‑regional coordination and reduced within‑region coordination.

### A recurrent model with dynamic synaptic efficacy captured efficient acquisition and flexible goal updating

**Fig 5:**
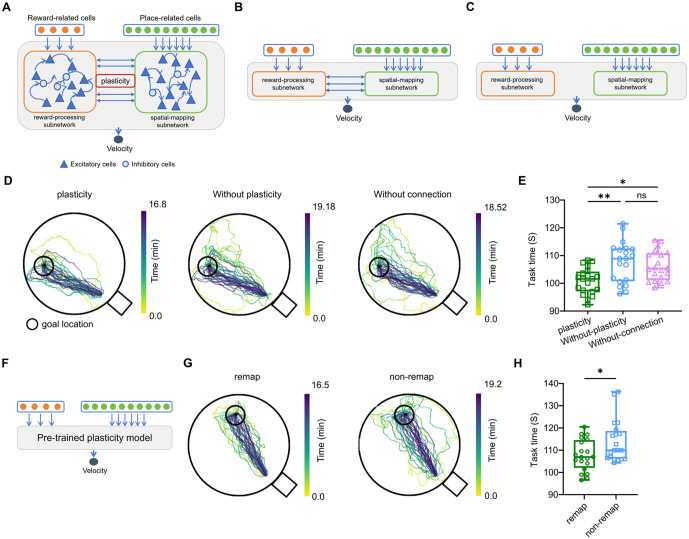
A recurrent model with dynamic synaptic efficacy captured key qualitative features of efficient acquisition and flexible goal updating. **(A–C)** Computational model architecture. This schematic illustrates the three versions of the recurrent neural network (RNN) used to simulate goal‑directed navigation. (A) The full model contains two interconnected subnetworks representing mOFC‑like goal‑related processing and dCA1‑like spatial processing, together with dynamic synaptic efficacy. (B) The no‑dynamic‑efficacy control model has an identical architecture to the full model but lacks dynamic synaptic efficacy. (C) The no‑cross‑connection control model is also identical in architecture but has no direct connections between the two subnetworks. **(D)** Example behavioral trajectories from a single trial for each of the three model variants: full model (left), no‑dynamic‑efficacy control (middle), and no‑cross‑connection control (right). **(E)** Quantitative comparison of the time required for each model variant to reach the goal. The full model reached the goal significantly faster than both control models (Friedman test, Dunn’s multiple‑comparisons test, full model vs. no‑dynamic‑efficacy control, *n* = 20 simulations, *Z* = 3.069, *p* = 0.0064; full model vs. no‑cross‑connection control, *n* = 20 simulations, *Z* = 2.716, *p* = 0.0198; no‑dynamic‑efficacy control vs. no‑cross‑connection control, *n* = 20 simulations, *Z* = 0.353, *p* > 0.9999). **(F)** Goal‑updating experiment schematic. This diagram illustrates how flexibility in the model was evaluated. A model that had been pre‑trained on a specific goal location was then assigned a new, distinct goal location, allowing assessment of whether prior training facilitated subsequent updating. **(G)** Example trajectories from a pre‑trained full model after goal relocation (left) and from a naive full model trained only on the new goal (right). **(H)** Comparison of the time required to reach the new goal for the pre‑trained full model vs. a naive full model without prior training (Mann–Whitney test, naive vs. pre‑trained, *n* = 20 simulations, *U* = 123, *p* = 0.0375). The underlying numerical data are provided in S5 Data.

Finally, to test whether the observed learning‑related coordination and behavioral flexibility could, in principle, be supported by a biologically motivated activity‑dependent synaptic efficacy rule, we developed a simplified RNN. The model consisted of two interconnected subnetworks representing mOFC‑like goal‑related processing and dCA1‑like spatial processing, with connections modulated by a dynamic synaptic efficacy rule [47] (Fig 5A). Under this rule, synaptic efficacy varies as a function of recent activity history rather than remaining fixed. We compared this full model to two control models: one with identical architecture but lacking the dynamic synaptic efficacy, and one where the two subnetworks were not directly connected (Fig 5B and 5C).

When simulated in a virtual goal‑directed task, the full model learned to navigate to the target location faster than either control model (Fig 5D and 5E). This suggests that dynamic synaptic efficacy contributed to more efficient learning in the model. Furthermore, the full model exhibited qualitatively similar flexibility to that observed in the rats. When a network that had been pre‑trained on one goal location was tasked with learning a new goal (Fig 5F), it did so more rapidly than a naive network (Fig 5G and 5H). Although these modeling results do not in themselves demonstrate that the same interaction rule directly drives the physiological changes observed in dCA1 and mOFC, they provide a simplified candidate framework linking learning‑related temporal coordination to adaptive goal‑directed navigation.

## Discussion

This study provides a multi‑scale investigation of the neural coordination patterns associated with flexible, goal‑directed spatial navigation. By combining simultaneous in vivo recordings, advanced signal analysis, and computational modeling, we show that learning‑related coordination between the mOFC and the dCA1 is closely associated with this cognitive function. Our key findings are as follows: (1) goal‑related reorganization was observed in both regions, with mOFC pyramidal neurons showing stronger learning‑related updating; (2) joint dCA1–mOFC population activity improved decoding relative to either region alone, consistent with complementary contributions; (3) oscillatory coordination between the two regions was state dependent and became stronger during navigation later in learning; and (4) learning‑related changes in short‑latency spike‑time coordination were linked to region‑specific neuronal reorganization and were further explored using a dynamic synaptic efficacy model.

### A working framework for complementary mOFC–dCA1 contributions to goal‑directed navigation

Our results motivate a refinement of the classical view that simply segregates the mOFC and the dCA1 into “value” and “space” modules, respectively [18,71,72]. We propose that these regions make complementary contributions to a distributed representation supporting goal‑directed navigation. In this framework, the dCA1 provides the stable, allocentric spatial scaffold necessary for localization (“where am I?”), consistent with its well‑documented role in forming cognitive maps. The mOFC, in turn, may update this map with information about the current behavioral objective (“where am I going?”), a role supported by our observation that its pyramidal neurons more flexibly remap to represent new goal locations [73–75]. Basu and colleagues [25] showed that OFC neurons can represent goal‑related variables such as distance‑to‑goal during navigation. Extending beyond this single‑region, largely static framing, our study shows that when goal locations change daily, mOFC shows stronger learning‑related updating of goal‑related coding toward the currently relevant goal, and this flexible goal representation is temporally coordinated with hippocampal dCA1. Across learning blocks, inter‑regional theta coherence and theta–gamma coupling strengthen, accompanied by learning‑dependent changes in short‑latency spike‑time coordination, together supporting an interpretation in which goal signals in mOFC interact with spatial mapping in dCA1 during flexible navigation.

The population decoding analysis provides convergent support for this complementary view. Specifically, the combined neural population predicted behavioral state more accurately than either region alone, indicating that behaviorally relevant information was more fully captured when activity from both regions was considered together. This finding is consistent with distributed coding across the two regions [76,77].

Although the present study did not include lesion or inactivation experiments, the involvement of dCA1 in this task is consistent with extensive causal evidence that hippocampal integrity is required for spatial learning and place navigation [78]. Likewise, prior studies have shown that mOFC contributes to outcome retrieval and expectancy‑guided behavior [79,80], and reversible OFC inactivation has been reported to disrupt hippocampus‑dependent spatial memory tasks [81]. More recent simultaneous‑recording work further supports the biological plausibility of coordinated hippocampal–orbitofrontal processing by showing theta‑modulated dynamics across these regions during cognitive behavior [73]. We therefore interpret the present findings as evidence that learning‑related mOFC–dCA1 coordination is associated with performance in the daily goal‑location task, while direct causal tests remain an important goal for future work.

### The role of oscillatory dynamics in state‑dependent communication

Oscillatory coordination has been proposed to structure inter‑areal communication by providing temporally organized windows for information transfer and learning‑related interactions [58,82,83]. In line with this framework, we found that mOFC–dCA1 coordination during navigation was stronger later in learning, manifested as increased theta‑phase synchronization and enhanced dCA1‑theta to mOFC‑gamma phase–amplitude coupling. Such learning‑linked rhythmic coordination is consistent with evidence that cross‑frequency coupling and long‑range synchronization track learning and support hippocampal and prefrontal computations [12,45,84].

In the orbitofrontal cortex, the Pennartz lab [85] reported that learning is accompanied by increased gamma‑band phase‑locking of action–outcome selective neurons, supporting the view that OFC computations are embedded in learning‑dependent temporal organization. We extend this line of work beyond local OFC dynamics by showing that mOFC rhythmic activity becomes temporally coordinated with hippocampal dCA1 as animals acquire a flexible spatial goal. Prior work has further shown that OFC theta and gamma dynamics track associative decisions and that experimentally perturbing OFC theta can impair reward‑based learning [29,86]. More recent studies have also emphasized coordinated theta‑organized activity across hippocampal–orbitofrontal or hippocampal–prefrontal circuits during task performance [30,87]. Our findings extend these results into the setting of flexible spatial goal updating with simultaneous single‑unit and LFP measures. We further relate these LFP‑level signatures to spike‑level measures of functional timing relationships: learning‑related increases (or decreases) in short‑latency spike‑time coordination across blocks were associated with stronger (or weaker) temporal relationships between mOFC and dCA1 neuronal populations. This provides a conceptual link between oscillatory coordination and learning‑related changes in spike‑time relationships, further supported by our dynamic synaptic efficacy model showing that such interaction rules can, in principle, account for improved navigation efficiency.

Together, our findings suggest that mOFC–dCA1 communication is state‑dependent rather than static. During navigation, enhanced theta coherence and theta–gamma coupling may reflect a regime in which hippocampal theta more strongly constrains the timing of mOFC activity. Such coordination could help align goal‑related and spatial information during ongoing navigation, although this interpretation remains to be tested directly [82]. This interpretation is consistent with work implicating long‑range theta coherence between hippocampus and prefrontal cortex in working memory and selective attention [84,88]. In contrast, the reduction of coupling at the goal may reflect a functional decoupling as computational demands shift toward reward consumption and outcome evaluation, processes in which local mOFC computations—reflected in its intrinsic gamma activity—may become more dominant [89,90]. Such dynamic coupling/decoupling may represent an efficient strategy for flexibly switching communication modes to match moment‑to‑moment cognitive demands [91].

### Learning‑related changes in short‑latency spike‑time coordination

Our study provides in vivo evidence consistent with learning‑related timing coordination as a potential contributor to this dynamic interaction. The selective increase in short‑latency coordination of dCA1‑Pyr and mOFC‑Pyr pairs suggests that learning is accompanied by more organized temporal coordination between the two structures [73]. The finding that this timing coordination is most prominent for neurons that remap to the new goal location suggests that this reweighting may preferentially support updating of goal‑related representations within the circuit [92,93]. Together, these learning‑associated changes in spike‑timing relationships may provide a more persistent component that could interact with the transient, state‑dependent oscillatory coupling observed during navigation.

Importantly, the pairwise timing analysis showed a region‑specific pattern: in mOFC, increased short‑latency coordination preferentially involved reorganization‑linked pyramidal neurons, consistent with selective updating of goal‑related population interactions and with prior evidence that orbitofrontal gamma‑band coordination is shaped by learning and action–outcome structure [85,86]. In dCA1, by contrast, non‑reorganization neurons showed a different pattern, contributing more often to increased cross‑regional coordination while being associated with decreased within‑region coordination. This dissociation suggests that learning retunes fine‑timescale coordination in a region‑specific manner, rather than through a uniform strengthening across the network. At the same time, these short‑latency interactions should be interpreted as measures of functional coordination rather than direct evidence of monosynaptic connectivity [70,94].

### Dynamic synaptic efficacy provides a candidate framework for adaptive navigation

Our computational modeling provides a candidate framework for interpreting how learning‑related temporal coordination might support flexible navigation, while also clarifying the limits of the present study. In the model, a recurrent architecture combining goal‑related and spatially structured subnetworks, together with time‑dependent synaptic efficacy, was able to capture key qualitative features of efficient initial acquisition and faster updating after a goal change. These simulations therefore show that temporally dependent interaction rules can, in principle, generate key qualitative features of the behavioral flexibility observed in the data.

Although dCA1 is unlikely to receive a direct monosynaptic input from mOFC, several indirect pathways could support the learning‑dependent temporal coordination we observe. One plausible route is via the lateral entorhinal cortex (LEC), which receives orbitofrontal inputs [95] and provides a major cortical interface to the hippocampus, conveying multimodal sensory information through direct entorhinal projections to CA1 [96,97]. In this framework, enhanced inter‑regional theta coordination could temporally align mOFC‑related signals (relayed via LEC) with hippocampal theta phase, creating a structured timing window that could facilitate learning‑related interactions in downstream circuits [98]. This pathway offers one plausible anatomical route through which the LFP‑level coupling results, the learning‑dependent changes in short‑latency spike‑time coordination, and the behavioral improvement captured by the dynamic synaptic efficacy model may be related.

At the same time, several important limitations remain. The present analyses do not by themselves establish the precise circuit pathway or synaptic architecture underlying dCA1–mOFC coordination, and the computational model was intended only as a simplified candidate framework rather than a biologically complete reconstruction. In addition, coordination between these regions is likely to depend on intermediary pathways and modulatory influences that were not resolved here. Finally, although the present findings identify robust learning- and state‑dependent coordination between dCA1 and mOFC, they do not directly establish that either region is causally necessary for performance in the daily goal‑location task. Future work combining pathway‑specific perturbations, large‑scale recordings, and more structured circuit models will be needed to determine how these distributed interactions are implemented mechanistically.

In conclusion, this study identifies a multi‑scale pattern of learning‑related coordination associated with flexible goal‑directed navigation. Our results suggest that adaptive behavior is accompanied by a reweighting of temporal coordination across dCA1 and mOFC, together with state‑dependent oscillatory coupling.

## Supporting information

S1 Fig Behavioral and locomotor controls for learning‑related oscillatory changes in dCA1 and mOFC. (**A**) Navigation duration during the first and second navigation epochs in Block 1 and Block 4. Navigation duration was significantly reduced in Block 4 relative to Block 1 (Mann–Whitney *U* test, Block 1 vs. Block 4, Navigation 1, *p* < 0.0001; Navigation 2, *p* < 0.0001). (**B**) Trial‑by‑trial average locomotor velocity across learning trials. (**C**) Mean locomotor velocity in Block 1 and Block 4. No significant difference was observed between blocks (Wilcoxon test, Block 1 vs. Block 4, *n* = 14 sessions, *W* = 41, *p* = 0.2166). (**D**) Distribution of navigation‑stage velocity in Block 1 and Block 4. Velocities from 0 to 50 cm/s were binned in 2 cm/s intervals and are shown as z‑scored counts, indicating comparable velocity distributions across blocks (Wilcoxon test, Block 1 vs. Block 4, *n* = 25 bins, *W* = −45, *p* = 0.5602). (**E**) Scatterplots showing the relationship between locomotor velocity and power spectral density (PSD) in dCA1 and mOFC for theta, low‑gamma, and high‑gamma bands. Power values were estimated in 200 ms windows and compared with the mean velocity in the same window; Pearson’s r and *P* values are indicated. (**F**) Estimated breakpoints from piecewise fits of trial‑wise oscillatory power in dCA1 and mOFC across frequency bands. (**G**) Pre- and post‑breakpoint slopes derived from the piecewise fits in dCA1 and mOFC (Multiple Wilcoxon matched‑pairs signed‑rank tests with FDR correction, adjusted *q* values: dCA1, pre‑slope vs. post‑slope, *n* = 14 sessions, *W* = −97, *q* = 0.0008; mOFC, pre‑slope vs. post‑slope, *n* = 14 sessions, *W* = −79, *q* = 0.0054). (**H**) Estimated breakpoint from the piecewise fit of behavioral trajectory length across trials. (**I**) Pre- and post‑breakpoint slopes for behavioral trajectory length. Together, these analyses suggest that learning‑related changes in oscillatory activity were not readily explained by differences in locomotor speed and unfolded alongside early improvement in behavioral performance (Wilcoxon test, post‑slope vs. pre‑slope, *n* = 14 sessions, *W* = 105, *p* = 0.0001). The underlying numerical data are provided in S6 Data.
(TIF)

S2 Fig Decoding performance following partial replacement of mOFC neurons with dCA1 neurons. (**A**–**C**) ROC‑AUC for decoding behavioral stage, learning blocks during the Goal epoch, and learning blocks during the Navigation epoch after replacing 0%, 10%, 20%, 30%, or 40% of mOFC neurons with dCA1 neurons (Friedman test, Dunn’s multiple‑comparisons test, *p < 0.05, **p < 0.01, ***p < 0.001, ****p < 0.0001). The underlying numerical data are provided in S7 Data.
(TIF)

S3 Fig Stage‑dependent gamma power and dCA1–mOFC theta–gamma coupling during Goal and Navigation epochs. (**A**) Low- and high‑gamma PSD in dCA1 and mOFC during the Goal epoch (Wilcoxon test, Low *γ*, mOFC versus dCA1, *n* = 14 sessions, *W* = −105, *p* = 0.0001; High *γ*, mOFC versus dCA1, *n* = 14 sessions, *W* = 85, *p* = 0.0052). (**B**) Low- and high‑gamma PSD in dCA1 and mOFC during the Navigation epoch (Wilcoxon test, Low *γ*, mOFC versus dCA1, *n* = 14 sessions, *W* = −105, *p* = 0.0001; High *γ*, mOFC versus dCA1, *n* = 14 sessions, *W* = 93, *p* = 0.0017). (**C**) PLV between dCA1 and mOFC low- or high‑gamma activity during Goal and Navigation epochs in Block 1 and Block 4 (Multiple Wilcoxon matched‑pairs signed‑rank tests with FDR correction, adjusted *q* values: Low *γ*, Navigation, Block 1 versus Block 4, *n* = 14 sessions, *W* = −75, *q* = 0.0335; Low *γ*, Goal, Block 1 versus Block 4, *n* = 14 sessions, *W* = 35, *q* = 0.2987; High *γ*, Navigation, Block 1 versus Block 4, *n* = 14 sessions, *W* = −93, *q* = 0.0017; High *γ*, Goal, Block 1 versus Block 4, *n* = 14 sessions, *W* = 51, *q* = 0.0600). (**D**) MI of dCA1 theta–mOFC gamma coupling during Goal and Navigation epochs (Multiple Wilcoxon matched‑pairs signed‑rank tests with FDR correction, adjusted *q* values: Low *γ*, Goal versus Navigation, *n* = 14 sessions, *W* = −76, *q* = 0.0281; High *γ*, Goal versus Navigation, *n* = 14 sessions, *W*=−3, *q* = 0.9610). (**E**) n:m PLV spectra between dCA1 theta and mOFC low- or high‑gamma activity during Goal and Navigation epochs (Wilcoxon test, low‑gamma, Goal versus Navigation, *n* = 14 sessions, *W* = 53, *p* = 0.1040; high‑gamma, Goal versus Navigation, *n* = 14 sessions, *W* = 55, *p* = 0.0906). The underlying numerical data are provided in S8 Data.
(TIF)

S4 Fig Field size and distance between firing peaks with goal location in mOFC and dCA1. (**A**) Comparison of 80%-peak firing‑field area, defined as the number of spatial bins with firing rates exceeding 80% of the peak firing rate, for pyramidal cells and interneurons in mOFC and dCA1 during pre‑probe and post‑probe sessions (Friedman test, Dunn’s multiple‑comparisons test, left, dCA1 Pyr versus dCA1 Int, *n* = 14 sessions, *Z* = 2.826, *p* = 0.0283; right, mOFC Int versus dCA1 Pyr, *n* = 14 sessions, *Z* = 2.965, *p* = 0.0182). (**B**) Distances from the peak firing location to the previous and current goal locations. In pre‑probe sessions, cells classified as previous goal‑related had firing peaks closer to the previous than the current goal location, whereas in post‑probe sessions, cells classified as current goal‑related had firing peaks closer to the current than the previous goal location (Wilcoxon test, pre‑probe, current versus previous, *n* = 81 units, *W* = 1,983, *p* < 0.0001; post‑probe, current versus previous, *n* = 101 units, *W*=−4,819, *p* < 0.0001). The underlying numerical data are provided in S9 Data.
(TIF)

S5 Fig Reorganization‑related enrichment in within‑region pyramidal–pyramidal pairs with increased or decreased short‑latency coordination in dCA1 and mOFC. (**A**) Schematic of the analysis of within‑region pyramidal cell to pyramidal cell (Pyr–Pyr) interactions. (**B**) dCA1 Pyr–Pyr pairs. Counts of neurons classified as reorganization or non‑reorganization neurons among Pyr–Pyr pairs showing increased or decreased short‑latency coordination across learning (Wilcoxon test, dCA1, Pyr–Pyr, Reorganization, Decreased versus Increased, *n* = 14 sessions, *W* = 21, *p* = 0.2539; dCA1, Pyr–Pyr, non‑Reorganization, Decreased versus Increased, *n* = 14 sessions, *W* = 64, *p* = 0.0020). (**C**) mOFC Pyr–Pyr pairs. Counts of neurons classified as reorganization or non‑reorganization neurons among Pyr–Pyr pairs showing increased or decreased short‑latency coordination across learning (Wilcoxon test, mOFC, Pyr–Pyr, Reorganization, Decreased versus Increased, *n* = 14 sessions, *W*=−57, *p* = 0.0088; mOFC, Pyr–Pyr, non‑Reorganization, Decreased versus Increased, *n* = 14 sessions, *W*=−43, *p* = 0.0547). The underlying numerical data are provided in S10 Data.
(TIF)

S6 FigTheta phase coupling and sharp‑wave ripple occurrence in dCA1 and mOFC during Goal and Navigation epochs.(**A**) Representative examples of theta phase‑coupled neurons, shown as spike‑phase histograms and polar plots of firing probability across theta phase. (**B**,**C**) Number of theta phase‑coupled neurons and theta phase concentration (κ) in dCA1 (B) and mOFC (C) during the Goal epoch, compared between Block 1 and Block 4 (B: Wilcoxon test, dCA1, Goal, left, Block 4 versus Block 1, *n* = 14 sessions, *W*=−5, *p* > 0.9999; right, Block 4 versus Block 1, *n* = 14 sessions, *W* = 3, *p* = 0.8438; C: Wilcoxon test, mOFC, Goal, left, Block 4 versus Block 1, *n* = 14 sessions, *W*=−5, *p* > 0.9999; right, Block 4 versus Block 1, *n* = 14 sessions, *W* = 15, *p* = 0.1563). (**D**,**E**) Number of theta phase‑coupled neurons and theta phase concentration (κ) in dCA1 (D) and mOFC (E) during the Navigation epoch, compared between Block 1 and Block 4 (D: Wilcoxon test, dCA1, Navigation, left, Block 4 versus Block 1, *n* = 14 sessions, *W* = **−**55, *p* = 0.0020; right, Block 4 versus Block 1, *n* = 14 sessions, *W*=−9, *p* = 0.6953; E: Wilcoxon test, mOFC, Navigation, left, Block 4 versus Block 1, *n* = 14 sessions, *W*=−38, *p* = 0.0283; right, Block 4 versus Block 1, *n* = 14 sessions, *W* = 7, *p* = 0.4375). (**F**) Representative example of a sharp‑wave ripple (SWR) event shown in the raw LFP (1–400 Hz) and ripple‑band filtered signal (150–250 Hz). (**G**) Number of SWRs detected during Goal and Navigation epochs across Blocks 1–4 (Multiple Wilcoxon matched‑pairs signed‑rank tests with FDR correction, adjusted *q* values, *q < 0.05, **q < 0.01, ***q < 0.001, ****q < 0.0001). The underlying numerical data are provided in S11 Data.(TIF)

S7 Fig Histological verification of recording sites in mOFC and dCA1. (**A**) Representative coronal sections from example animals showing electrode tracks and final recording locations in mOFC and dorsal CA1. Black lines indicate electrode tracks.
(TIF)

S1 Table Session‑wise counts of significant and total cross‑regional mOFC‑to‑dCA1 pyramidal–pyramidal pairs in the reorganization and non‑reorganization groups. (CSV)

S2 Table Session‑wise counts of significant and total cross‑regional dCA1‑to‑mOFC pyramidal–pyramidal pairs in the reorganization and non‑reorganization groups. (CSV)

S3 Table Session‑wise counts of significant and total within‑regional mOFC pyramidal–pyramidal pairs in the reorganization and non‑reorganization groups. (CSV)

S4 Table Session‑wise counts of significant and total within‑regional dCA1 pyramidal–pyramidal pairs in the reorganization and non‑reorganization groups. (CSV)

S1 Data Raw data underlying graphs found in Fig 1. (XLSX)

S2 Data Raw data underlying graphs found in Fig 2. (XLSX)

S3 Data Raw data underlying graphs found in Fig 3. (XLSX)

S4 Data Raw data underlying graphs found in Fig 4. (XLSX)

S5 Data Raw data underlying graphs found in Fig 5. (XLSX)

S6 Data Raw data underlying graphs found in S1 Fig. (XLSX)

S7 Data Raw data underlying graphs found in S2 Fig. (XLSX)

S8 Data Raw data underlying graphs found in S3 Fig. (XLSX)

S9 Data Raw data underlying graphs found in S4 Fig. (XLSX)

S10 Data Raw data underlying graphs found in S5 Fig. (XLSX)

S11 Data Raw data underlying graphs found in S6 Fig. (XLSX)

## Abbreviations

AUC: area under the curve
CCGs: cross‑correlograms
dCA1: dorsal CA1
FDR: false discovery rate
ICA: Independent Component Analysis
ISI: inter‑spike interval
LEC: lateral entorhinal cortex
LFP: local field potential
MI: modulation index
mOFC: medial orbitofrontal cortex
PCA: Principal Component Analysis
PLV: phase-locking value
RNN: recurrent neural network
ROC: receiver operating characteristic
ROIs: regions of interest
RSS: residual sum of squares
STSP: short‑term synaptic plasticity
SWR: sharp‑wave ripple
